# How Cytoskeletal Disorders Contribute to Errors in the Chromosomal Segregation of Oocytes and Cleavage Stage Embryos

**DOI:** 10.3390/jdb13040043

**Published:** 2025-12-02

**Authors:** Stefka Delimitreva, Irina Chakarova

**Affiliations:** Department of Biology, Medical University of Sofia, 2 Zdrave Street, 1431 Sofia, Bulgaria; i.chakarova@medfac.mu-sofia.bg

**Keywords:** oocyte, meiosis, fertilization, preimplantation embryo, aneuploid embryo

## Abstract

Observations of the processes of oogenesis, fertilization, and the earliest embryonic development have given us the opportunity to estimate the importance of chromosomal distribution errors for the success of mammalian reproduction. It is now known that in the large volume of oocytes, zygotes and the first embryonic cells, the rearrangement of chromatin is associated with a complex rearrangement of cytoskeletal structures, which creates specific problems. This review discusses two main issues critical to the success of early embryos: Why oocyte meiosis is too frequently wrong in chromosomal segregation? Why the first zygotic mitoses are too frequently wrong in chromosomal segregation? We concluded the following: (1) The main cytoskeletal defects that disturb oocyte meiosis are a problematic connection between cytoskeleton and nucleoskeleton, unsuccessful movement of the spindle to the oocyte periphery, unstable anchoring of the spindle to oolemma, and deviations in meiotic spindle morphology; (2) The main cytoskeletal defects that disturb pronuclear unification are nonfunctional male centriole, unsuccessful forming of microtubule aster around the sperm centrosome, problematic movement of the two pronuclei towards each other and inappropriate contacts between centrosomes, microtubules and nuclear pore complexes; (3) Cytoskeletal defects that disturb zygote mitosis are unsuccessful forming of bipolar mitotic spindle, non-synchronized congression of maternal and paternal chromosomes, and unsuccessful attachment of kinetochores to microtubules.

## 1. Introduction

The beginning of the study of the earliest stages of mammalian embryogenesis was set by Carl Hartmann. In 1931, he first described bovine preimplantation embryos obtained by washing the oviducts [1]. Oviduct washing to receive eggs and preimplantation embryos is applicable to mammals with easily predictable or controllable estrus. This approach was used to gather data on fertilization and early embryogenesis in ungulates, rodents, rabbits [2,3,4,5]. These early studies became the scientific basis for the development of in vitro fertilization (IVF) techniques and other approaches of assisted human reproduction, in addition to initiating fundamental scientific research of human oogenesis and early embryogenesis. Now, almost a hundred years after the beginning of embryological observations, some details of these processes are still not fully understood.

One of the important questions that is still waiting for its full explanation is, what are the molecular reasons for the compromised quality of oocytes and early embryos that lead to reduced fertility?

The available data show that errors in chromatin rearrangement, which is mediated by the cytoskeleton, have a decisive contribution to this.

– The rearrangement of maternal chromosomes during the final stages of meiosis is prone to errors because it requires complex cytoskeletal interactions due to the large volume of oocytes.
– During the zygotic stage, cytoskeletal elements are involved both in the movement of pronuclei towards each other and in the transformation of their chromatin into dense clusters that must be localized in their boundary zones.
– Errors can also arise from the lack of alignment of the two spindles, from which the first mitotic spindle of the zygote is formed.

In vitro fertilization was developed as an approach to treating human infertility. Soon after its clinical introduction, there was disappointment with the relatively limited success rate of the method. Initially, it was assumed that this was the result of suboptimal conditions of in vitro cultivation systems, hormonal stimulation, and egg aspiration techniques. Therefore, efforts to improve success rates were aimed at optimizing these factors. Nowadays, the conditions under which in vitro fertilization takes place are as close as possible to those of natural conception, but the results are far from perfect—according to presented at the European Society of Human Reproduction and Embryology (ESHRE) 41st Annual Meeting, 1 July 2025 [6], clinical pregnancy rates remained stable between 2021 and 2022. For IVF, the rate per aspiration/thawing was 26.3% in 2021 and 25.8% in 2022, while the rate per transfer was 33.5% and 32.7%, respectively. These data show that the potential for improving the success rate of assisted reproduction, although not yet exhausted, is limited. Therefore, the decisive factors for reproductive success are some natural characteristics of the embryo. We now know that such a reason is the natural tendency of oocytes and embryos at the cleavage stage to make errors in chromosomal distribution. In other words, chromosomal errors are the most important cause of failed conception.

The errors that lead to aneuploid embryos can be meiotic as well as mitotic. A large number of in vitro studies in recent decades have shown that a high proportion of mammalian embryos (for example, 50–70% of human embryos) at the cleavage stage have an incorrect distribution of chromosomes [7,8]. This is due to both errors made in the gametes (with the highest probability in anaphase I and anaphase II of oocyte meiosis) and errors in the first mitoses of the zygote [9]. Most aneuploid in vitro produced embryos do not reach successful implantation in the uterus, and even if they survive to this stage, they do not survive until the end of pregnancy [8]. Now we can confidently say that aneuploidy in preimplantation embryos is the main cause of miscarriages. This fact leads us to the idea that the level of fertility in mammals depends on the correctness of the chromosomal distribution in the last stages of oocyte meiosis and the first embryonic mitoses [9,10,11,12]. Since it was noticed that in human in vitro produced oocytes and early embryos the level of numerical chromosomal errors is unexpectedly high, this fact is considered one of the main reasons for unsuccessful in vitro (possibly in vivo) fertilization.

## 2. Why Oocyte Meiosis Is Too Frequently Wrong in Chromosomal Segregation?

### 2.1. In Oocyte Meiosis, the Control of the Cell Cycle Is Loosened

The meiosis of oocytes in mammals begins in the embryonic ovary, but there the process stops when the end of the prophase of the first meiotic division (prophase I) is reached. The restoration of meiosis in individual eggs begins at puberty. That is coupled with a complex chain of hormonal and paracrine stimuli and requires precise synchronization of follicle and oocyte maturation. This complexity leads to defects in the meiotic process. That is supported by clinical data—for some categories of patients, it has been proven that the reduced chance of conception is due to an incorrect distribution of chromosomes in oocyte meiosis [13].

It has been shown that numerical chromosomal errors in oocytes occur mainly during the metaphase-anaphase transition of the first meiotic division. The checkpoint responsible for the accuracy of this transition in the meiotic spindle is called the meiotic spindle checkpoint [14,15]. This checkpoint should stop the development of oocytes with errors in chromosomal distribution between the oocyte and the polar body, preventing the completion of their meiotic maturation, their eventual fertilization and the creation of an-euploid embryos.

During mitosis, a similar checkpoint acts, allowing or prohibiting the metaphase-anaphase transition, evaluating the correct arrangement of microtubules in the mitotic spindle and the correctness of their connections with chromatin. The control of meiotic division has been much less studied. Not all the facts related to mitosis checkpoints can be directly attributed to meiosis, in particular because of the following facts: in oocytes and spermatocytes homologous chromosomes conjugate and form bivalents; sister chromatids have common kinetochores and during anaphase I do not separate from each other; in oocyte meiosis, things are further complicated due to the years in which the process is arrested at prophase I (the so-called dictyate stage), the complex regulation through pituitary and ovarian hormones, the participation of somatic follicle cells in it and, last but not least, the peculiarities of cytoskeletal interactions. Although there are no direct illustrations for some details of the processes, the general picture of the course and control of cytoskeletal and chromatin transformations during oocyte maturation have been clarified.

Now we know that oocyte meiosis allows for irregular chromosomal distribution due to reduced efficiency of the spindle checkpoint, and this is an important cause for aneuploidy in oocytes. On the other hand, the relatively high percentage of mosaic preimplantation embryos [16] shows that probably the control of the cell cycle is imperfect also in the first mitoses of the zygote. Therefore, the disturbances in both oocyte meiosis and the first mitoses of the zygote contribute to numerical chromosomal errors in preimplantation embryos.

### 2.2. During Oocyte Meiosis, the Driving Force of the Cytoskeleton Is Transmitted from the Cytoplasm to the Nucleus

During the prolonged prophase of the first meiotic division, homologous chromosomes are connected by synaptonemal complexes, which provides them with the correct position for crossover. For a synapse to be carried out between homologous chromosomes, they must first move towards each other. Their movement is controlled by cytoskeletal forces, in which complexes of proteins referred to as LINC (linker of nucleoskeleton and cytoskeleton complex) are involved [17]. They contain proteins with DNA binding domains that recognize specific telomere sequences. The role of LINC complexes is to attach chromosomal telomeres to the nuclear envelope. In this way, they make a connection between the cytoplasmic cytoskeleton and the nucleoskeleton [18]. Thus, a force generated in the cytoplasm is exerted on the chromosomes. Evidence of this is the fact that mice with defective proteins included in LINC (SUN-1 and KASH5) have been shown to be infertile due to an inability to complete their meiosis [19].

### 2.3. Participation of Various Elements of the Cytoskeleton in the Assembly and Movement of the Meiotic Spindle

**Figure 1:**
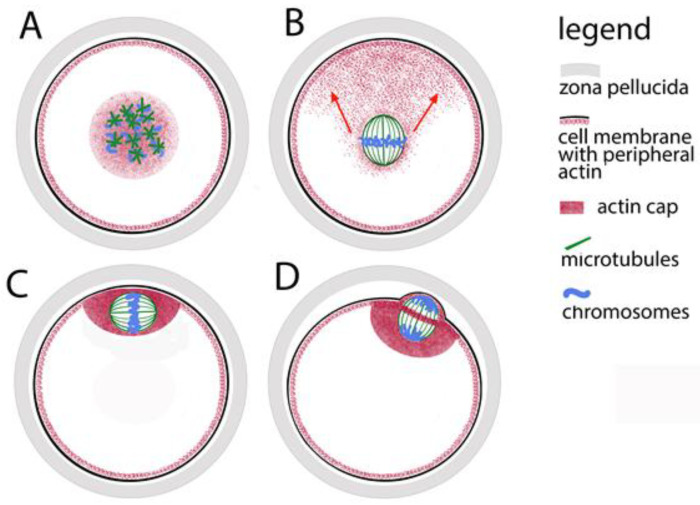
Assembly of the meiotic spindle in mammals and its relocation to the oocyte periphery. (**A**) Formation of tubulin stars on the chromosomes. (**B**) Fibrillar actin pulls the spindle towards the oolemma (oocyte cell membrane); the direction is marked with red arrows. (**C**) The actin cap anchors the spindle to the oolemma. (**D**) During polar body extrusion, the dividing groove passes through the spindle equator.

In practice, the leading role in the organization of the meiotic spindle is played by the chromatin of the oocytes. Proteins closely associated with chromosomes guide the polymerization of tubulin. As a result of hormonal stimulation, during the awakening of meiosis, the chromatin of the oocyte prepares for the transition from dictyate to metaphase I. At this stage of its maturation, a nucleus is still observed in the oocyte. In the highly specialized literature, it is usually not referred to as a nucleus but is traditionally called a germinal vesicle (GV). At this stage, the tubulin is predominantly located around it. In the transition to metaphase I, the nuclear envelope disintegrates, the tubulin comes into contact with the chromatin, and many small microtubular asters form around the chromosomes (Figure 1A). Together with the continued thickening and shortening of chromosomes, these asters grow, approach each other and fuse to form the meiotic spindle. This way, the poles of the spindle in oocytes are formed without the participation of centrioles. The time for fusion of tubulin asters into a bipolar spindle was measured for human oocytes [20]—it takes about 7 h. Initially, the spindle is assembled in the center of the oocytes (Figure 1B), and then the fibrillar actin moves it to the periphery. Eventually the meiotic spindle is located under the membrane (Figure 1C). The purpose of this displacement is to provide an asymmetric separation of the cytoplasm (the dividing groove in cytokinesis passes through the equator of the spindle (Figure 1D). Thus, after anaphase I and telophase I, a haploid chromosome set is separated into a miniature polar body with minimal loss of cytoplasm from the oocytes.

Yoshida and co-authors (2024) report that in mouse eggs, in the absence of centrosomes in oocytes, the creation of two spindle poles is guided by kinetochores. While the attachment of microtubules to kinetochores in early prometaphase is not stabilized, the arrangement and crosslinking of microtubules is controlled by the protein kinases MPS1, localized in kinetochore complexes. Inhibition of this kinase leads to a delay in the transition from early to late prometaphase, to problematic fixation of the two poles of the spindle, and to an increase in the wrong interactions between microtubules and chromosomal kinetochores [21,22]. Wu and co-authors describe in detail the process of bipolar spindle formation in human oocytes [23]. According to their results, the formation of the first meiotic spindle in human oocytes starts with accumulation of kinetochore-nucleated microtubules that initially form multipolar intermediates. Finally, a bipolar spindle is established. The subsequent reorganization to obtain only two poles requires between 7 and 9 h.

The chromatin-tubulin interaction is leading in the formation of the spindle, but the length of microtubules is limited—they can effectively bind to chromosomes only in cells whose diameter does not exceed 30 μm. In other words, the chromosome capture is efficient in small cells but may fail in cells with large nuclear volumes such as animal oocytes. That was proved by observation of growing microtubules and computer simulations of spindle assembly [24]. Therefore, microtubules would not successfully cope with the process described above in too large volume of ooplasm without the help of more dynamic actin microfilaments. The microfilaments are responsible for the initial movement of the chromosomes in a position convenient for assembling a spindle. It has been proven that even during the pachytene stage of prophase I, the telomere parts of chromosomes indirectly contact the actin network that surrounds the nucleus [25,26]. At the nuclear envelope breakdown, this network contracts, keeping the chromosomes close together, and thus mediates the convergence of the microtubule stars, the progenitors of the spindle (Figure 1A). In the subsequent stages of meiosis, microtubules remain closely related to actin microfilaments and their motor protein myosin. Thus, around the familiar spindle made up of microtubules, actin forms a secondary spindle of microfilaments [27]. The secondary actin spindle remains connected to the common actin network which reaches the cell membrane (Figure 1B). While the spindle is still located in the center of the oocyte, in the periphery opposite to one of its poles, a cluster of microfilaments is observed—so-called “oocyte actin cap” is formed (Figure 1C). With the help of myosin concentrated around the spindle poles, the clustered microfilaments direct the movement of the spindle to the periphery. The actin cap anchors the spindle under the cell membrane where it completes its first meiotic division, extrudes the first polar body and, reaching metaphase II, waits for the egg at fertilization.

The dynamics of the microfilaments is also responsible for the separation of the polar bodies. The spindle, whose axis during metaphase I is parallel to the oolemma, rotates so that during anaphase I and telophase I one pole is as close as possible to the membrane (Figure 1D). An actin ring is formed around the equator of the telophase spindle, which, narrowing, separates the polar body from the oocyte. Recently, another role of fibrillar actin in the oocyte has been proven—in mouse models, it has been found that the microfilaments in the meiotic spindle support the formation of the kinetochore fibers of the spindle and thus help stabilize the binding of chromosomes to the spindle and the correct chromosomal segregation [28].

Investigating the cytoskeletal dynamics mouse oocytes, our working group managed to show that before the breakdown of the nuclear envelope, the localization of cytoplasmic intermediate filaments (cytokeratins and vimentin) coincides with that of fibrillar actin, then cytokeratins and vimentin concentrate around the chromatin, and after the formation of the meiotic spindle, they remain connected to the chromosomes, but also form a diffuse layer around the spindle [29]. This shows that cytoplasmic intermediate filaments have a role both in increasing the permeability of nuclear membranes to provide access for the cytoplasmic cytoskeleton to the interior of the nucleus, and in the movement of chromosomes to ensure their contact with microtubules.

In addition, we also investigated the dynamics of nuclear intermediate filaments at the described stages. In investigated mammals (rats and mice), during meiosis, alternative splicing expresses period-specific lamins (e.g., lamin C2 from the A-lamin group). They provide specific cross-linking of the lamina, which aids in contact with telomeres and chromosomal movement [30]. We found that type A/C lamin is localized around the spindle, and type B lamin binds to microtubules [31]. From this we can assume that nuclear intermediate filaments also play a role in chromatin rearrangement during oocyte meiosis and that nuclear lamina defects are another potential cause of chromosomal re-arrangement failure.

### 2.4. Various Deviations in Meiotic Spindle Morphology Are Possible

Members of our working group have been studying cytoskeletal structures in mouse and primate (common marmoset *Callithrix jacchus*) oocytes for more than twenty years [32,33,34,35]. In both orders of mammals, we observed similar defects in tubulin and actin structures, which can be described in the following phenotypic classes: normal spindles, too large overall spindle sizes, presence of more than two poles, too wide poles, disorientation of microtubules or no spindle at all. In some cases, we also observed more than one spindle in the oocyte—one main spindle containing most of the chromosomes and a small extra spindle built around a small separate group of chromosomes.

The size of the meiotic spindle is the result of the equilibrium between the polymerization and depolymerization of the tubulin, which take place at the equator and the poles of the spindle. Microtubules are lengthened until the connection between them and a chromosomal kinetochore is established [36]. If for some reason this connection cannot be stabilized, the microtubules are excessively lengthened. The factors responsible for stabilizing the length of the spindle are kinesins localized between microtubules in the pole region and chromokinesins that connect microtubules to kinetochore protein complexes [35]. Therefore, excessive spindle growth may result from defects in the action of kinesins, chromokinesins and kinetochore proteins. Chromokinesin dysfunction can also be the cause of poor pole focusing, the formation of more than two poles, as well as the disorientation of microtubules in general.

The abnormally wide poles are probably the result of problems in the cooperative action of gamma-tubulin, pericentriolar material, and motor proteins (dynein) that are located in the polar regions. The role of these proteins in mitosis is to keep the ends of microtubules close together at the poles, as well as to connect the poles of the spindle with the actin cytoskeleton. In some cases, with extremely wide poles, the width of the spindle may exceed its length. In our research, we found that excessive pole width is not associated with disturbances in the chromosomal arrangement of the equator. This can be explained by premature stabilization of the length of the microtubules—it seems that in case of too short and wide spindles, this stabilization occurs before the final collection of microtubules at the poles.

In the first meiotic division, multipolar spindles may be the result of errors in the initial assembly of the spindle, but they may be the result of a transformation of a bipolar spindle into a multipolar spindle. This is the result of the instability of the first meiotic spindle—it often fails to stabilize and can transform into a globular or multipolar structure, which to-tally confuses chromosomal segregation [37].

As mentioned, oocyte chromosomes are the platforms on which the organization of tubulin asters begins, from which the spindle is subsequently assembled. The appearance of an additional small spindle along with the main one is evidence of the leading role of chromatin in the organization of microtubules—even single chromosomes are capable of building around themselves a bipolar structure similar to a spindle.

In some oocytes that stopped in metaphase I, we observed a visibly normal chromosome set located as expected under the membrane, but in the absence of any spindle [33]. Such a situation can be explained by the action of the secondary actin spindle—if for some reason there is no tubulin polymerization, only an actin spindle is assembled around the chromosomes after the disintegration of the nuclear envelope. This actin spindle carries the chromosomes to the cortical zone. Actin microfilaments are more dynamic while they are farther away from the chromosomes and more stable near them [33]. Therefore, cortical actin is able to hold chromosomes under the oolemma and without the participation of microtubules. Of course, in such cases, meiosis does not continue to anaphase segregation of chromosomes.

### 2.5. Complex Control over the Arrangement of Chromosomes in the Meiotic Spindle

During meiosis, chromosomal kinetochores undergo changes that are part of a cascade of reactions controlled by altering the activity of MPF (meiotic promoting factor). During prophase I and prometaphase I, kinetochores are not active. Kinetochore elements are present but need to mature through post-translational changes. The kinetochores become active and ready to bind to microtubules just before the onset of metaphase I, when the chromosomes are already located near the equator of the spindle [38]. Before this moment, microtubules are connected to the chromosomal arms by motor proteins of the class of chromokinesins (in humans they are referred to as Kid). Kid motors direct the movement of chromosomes by pushing their arms in the opposite direction to the poles. The force generated is called the “polar wind” for “polar ejection force” [39]. This force acts for several hours, at the end of which the chromosomes oscillate around the equator. The polar wind also acts in mitosis, but problems in its action damage meiosis more seriously, because in meiosis the non-kinetochore interactions between tubulin and chromatin have a greater relative contribution to the direction of chromosomes towards the equator. For the final arrangement of the chromosomes at the equator (chromosomal congression) and the establishment of metaphase, another force is needed: microtubules bind the already active kinetochores, another type of chromokinesins (in humans they are referred to as Kif) generate a force by which the microtubules pull each chromosome simultaneously to both poles [40].

It is known that incorrect attachment of chromosomes during mitosis is corrected before the final assembly of chromosomes and the final formation of the metaphase plate. In oocyte meiosis, however, corrections can also be performed after the onset of metaphase. At this point, single kinetochores connected to microtubules at both poles of the spindle are observed. The oocyte is given the opportunity to eliminate these errors until the metaphase chromosomal configuration is finally stabilized [41]. A signal for the presence of such errors is given by the tension between homologous kinetochores. The transformation of the wrong kinetochore attachments into regular attachments to one of the spindle poles is under the control of the Aurora A and Aurora B/C kinases, located at the poles and around the kinetochores, respectively [42].

Investigating marmoset oocytes matured in vitro, we have noticed that in some of them that failed to mature (to reach metaphase II), the chromosomes are entirely located inside the spindle, i.e., in them the microtubules are still connected not to the kinetochores, but to the chromosomal arms. Probably, in these cases, readiness for the final arrangement of metaphase I and transition to anaphase I was not achieved due to immaturity of the kinetochores or inadequate action of chromokinesins. This situation is observed more often in large and multipolar spindles and those with wide poles than in spindles of normal shape [33]. In some cases, the kinetochores are activated and the chromosomal arms are outside the spindle, but the chromosomes are not aligned at the equator. In human mitosis, this may be the result of defects in the action of chromokinesins or kinetochore proteins. In mammalian meiosis, the presence of monovalents in the spindle of metaphase I leads to such an effect (for example, in mice) [43].

### 2.6. DNA Damages Contribute to Meiotic Arrest

Based on the described cases of failed in vitro maturation and the fundamental data of the process, it can be summarized that the problems that arose during the synchronous meiotic remodeling of chromatin and cytoskeleton have a multiple down-stream effect on subsequent meiotic events and are a major factor in the incomplete maturation of oocytes.

The primary cause of errors in chromatin maturation and the formation of cytoskeletal structures during meiosis may be genetic—the result of certain gene combinations and/or gaps in DNA repair. For example, this can be illustrated by the fact that mouse oocytes carrying a mutation in a mismatch-repair gene cannot form stable spindles. Most of their chromosomes are in the form of monovalents with one kinetochore, their spindles growing excessively and often form more than two poles [43]. The significance of variations in the genome in the formation of the meiotic spindle is also confirmed by the differences in the size and shape of the spindles recorded in different mouse strains [44]. At the same time, it should be taken into account that such differences may also be due to changes in culture conditions (in vivo or in vitro), hormonal stimulation or the composition of the culture medium. Considering the high degree of heterozygosity in humans, it can be assumed that individual genetic features affect the meiotic rearrangement of chromatin and cytoskeleton in different ways.

## 3. Why the First Zygotic Mitoses Are Too Frequently Wrong in Chromosomal Segregation?

### 3.1. During Fertilization, Maternal and Paternal Chromatin Must Be Organized in Clusters

Establishing the correct position of parental genomes should ensure their successful unification. This process is mediated by the synchronous action of many cellular structures—the elements of centrosomes, dynein, microtubules, and nuclear pore complexes. Due to its complexity, the process is very prone to errors.

**Figure 2:**
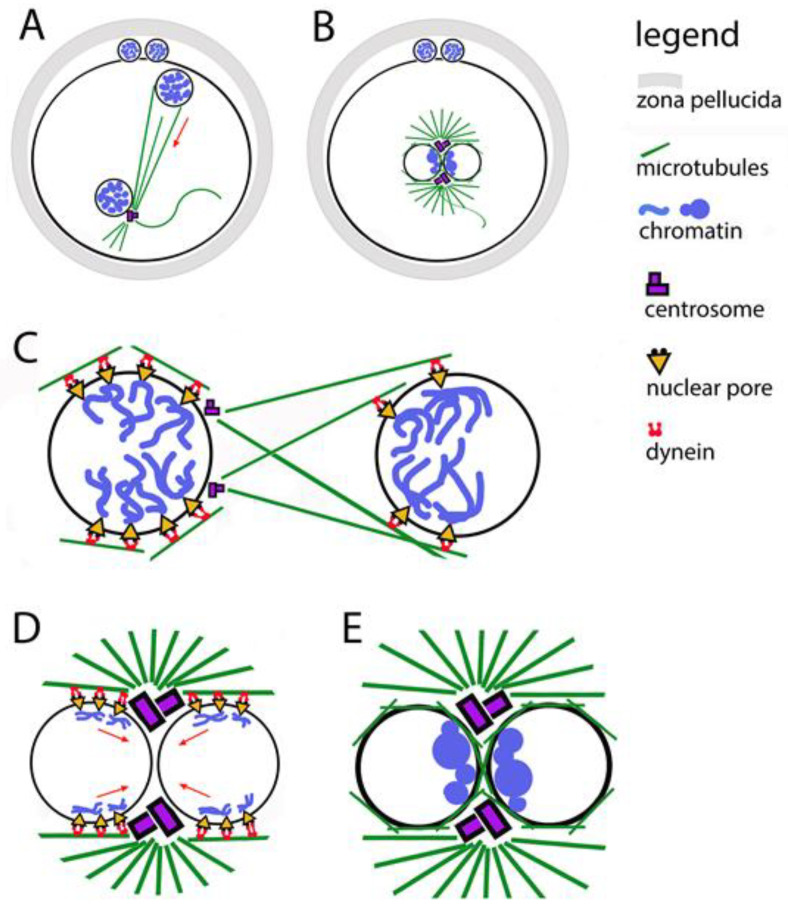
Relocation of the two pronuclei towards each other and clustering of their chromatin. (**A**) The male pronucleus is still associated with the axoneme of the sperm tail. The sperm centrosome organizes a tubulin star through which the pronuclei approach each other. The two polar bodies are located between the zona pellucida and the oolemma. (**B**) Contact between the two pronuclei in the center of the zygote; their chromatin is organized in clusters located in the boundary zone. (**C**) The figure illustrates the involvement of the two centrosomes, the microtubule star, dynein, and the nuclear pores in the movement of pronuclei. (**D**) The figure illustrates the involvement of the two centrosomes, the microtubule star, dynein, and the nuclear pores in the movement of chromatin clusters towards the contact zone between the pronuclei. (**E**) Close-up of the pronuclear contact; chromatin clusters located are in the boundary zone.

During fertilization, the maternal and paternal chromosomes are wrapped in nuclear envelopes, forming two haploid pronuclei. This happens in the periphery of the oocyte, which already has the right to be called a zygote (Figure 2). During the fusion of the two gametes, the formation of the male pronucleus and its movement into the ooplasm is assisted by actin nucleation [45]. At one pole of the male pronucleus, where a functioning centriole imported from the spermatozoon is localized, a microtubule aster is formed. Its fibers are used by pronuclei to approach each other while simultaneously moving towards the center of the zygote (Figure 2A). During the movement of the pronuclei, their chromosomes replicate, then they immediately begin to thicken, acquiring a prophase appearance and preparing to be included in the first mitotic spindle. The pronuclei make contact with each other, but do not merge. On the male pronucleus side, the centriole doubles, forming a fully mature centrosome, which in turn doubles (Figure 2B). Thus, each pronucleus has a centrosome, which allows the microtubule aster to transform into a bipolar spindle, while simultaneously destroying the envelopes of the pronuclei (Nuclear Envelope Breakdown, NEBD) [46].

The dynamics of the morphology of pronuclei during their movement towards each other and during their contact has been studied for decades, especially in assisted reproduction centers. For human zygotes, it has been found that the best chances of development have those in which male and female chromatin is highly condensed and located around the contact zone of the two pronuclei. Zygotes with dispersed chromatin or with chromatin clusters outside the contact zone have a much lower chance of developing into a blastocyst [47].

### 3.2. The First Meeting of the Maternal and Paternal Genomes Is on the Contact Surface of the Pronuclei

During the long fertilization process (about 24 h), the maternal and paternal chromosomes cluster in the contact zone of the pronuclei [47] (Figure 2B,E). Thus, the two chromosomal sets are arranged in a position convenient for their union. Microscopically, chromatin clusters (in some origins—nucleolar precursor bodies) look like nucleoli and are most often called that. No correlation has been proven between the number of nucleoli on both sides of the contact membranes and the probability of reaching the blastocyst stage. The position of these nucleoli and the time it takes for them to line up in the correct position are related to the developmental abilities of the embryo.

There is evidence to show that zygotes in which a cluster of chromatin is collected at the border of the two pronuclei is clearly observed have a greater chance of developing into blastocysts and less chance of giving rise to aneuploid embryos [48,49]. The time interval between the disintegration of the zygote nuclear envelope and its cytokinesis is also considered to be a prognostic sign [11]. In an analysis of videos of human preimplantation embryo development, it was found that in those that reach the blastocyst stage, the period from the breakdown of the nuclear envelopes of the pronuclei to the first division of the zygote is shorter than in embryos arrested at the cleavage stage.

A complete study of these relations in human embryos is difficult for ethical and legal reasons. It relies on data obtained from animal models. Relatively close to human embryos in terms of the mechanism of the earliest development are cattle embryos. They, like humans and unlike mice, have functional centrosomes introduced by the spermatozoa [50], similar duration of the first cell cycles [51], and similar levels of aneuploidy in preimplantation embryos [12,52].

### 3.3. Importance of Chromatin Cluster Formation for Chromosome Segregation

The pronuclei have a much larger diameter (up to 50 μm) compared to the nuclei of typical somatic cells, which requires a specific mechanism of microtubule-pronuclear interaction to guide their movement towards each other [53]. It is assumed that the organization of chromatin in clusters promotes microtubule-pronuclear contacts. This idea is supported by the fact that in about 70% of zygotes without clusters in the contact zone, the following defects are observed: asynchrony in NEBD; delays in the construction of the first mitotic spindle; delays in the arrangement of chromosomes in a metaphase plate; errors in the segregation of chromosomes during the first mitosis; individual chromosomes outside the spindle; lagging chromosomes during anaphase; formation of micronuclei. These defects are due to problematic contacts of the chromosomal arms and kinetochores with the microtubules. It has been observed that when pronuclei come into contact, chromosomes at the periphery of the chromatin clusters are more likely to lag during anaphase than those in the clusters. Anaphase lagging of chromosomes is observed much more often in preimplantation embryos than in somatic cells [54]. Therefore, in embryos at the cleavage stage, errors of chromosomal segregation are made much more often than at a later stage of development.

### 3.4. Centrosomes, Through Their Connection with Microtubules, Are Responsible for the Accuracy of Chromosome Segregation in the First Mitosis

Centrosomes are responsible for the successful arrangement of microtubules around pronuclei [55]. It is known that in zygotes in which the centrosomes are not in their correct position on the boundary surface of the pronuclei, the grouping of chromatin clusters takes longer and is more often unsuccessful [48]. A prerequisite for the correct chromosomal segregation between the first two blastomeres is the binding of chromosomes to centrosomes even before the disintegration of the nuclear envelopes of the pronuclei (Figure 2E).

In general, the transport of chromosomes to the centrosomes is mediated by the directed action of the motor protein dynein. In fertilization, this function of dynein is used to direct chromatin clusters to the centrosomes [56]. When the centrosomes double, the chromosomes cluster close to them on the contact surface of the pronuclei. To ensure the connection of chromosomes to dynein, nuclear pore complexes are used as adapters between the inside and outside of nuclear envelopes (Figure 2C,D). Thus, microtubules are involved in the process of organizing chromatin clusters even before the disintegration of nuclear envelopes. The data show that the orientation of chromatin to the boundary zone is completed before the contact of the pronuclei, and it is possible to be corrected after the contact [47]. It has been proven that when treating zygotes with substances that interfere with the polymerization of tubulin, in addition to blocking the migration of pronuclei to each other, the parental genomes fail to orient themselves correctly to each other.

### 3.5. The Adaptor Function of Nuclear Pore Complexes in the Localization of Chromatin Clusters

Nuclear pore complexes (NPCs) are embedded in the nuclear envelope. They mediate the connection between chromatin and cytoplasmic dynein [57]. This is valid for somatic cells, zygotes, and preimplantation embryos. Gavazza and coauthors [47] report that NPCs are unevenly distributed across pronuclear envelopes, with the most numerous being around chromatin clusters (Figure 2C,D). The place of concentration of nuclear pores coincides with the localization of dynein, as well as with the place of accumulation of microtubules between the two pronuclei.

In studies of mouse zygotes, the nucleoporin Elys was identified, which binds directly to chromatin, helping the pores to bind to chromatin and form clusters at one pole of each pronucleus [58]. These processes start at the beginning of the pronuclear migration. In the polarization of the female pronucleus, the pores and chromatin clusters are oriented towards the male pronucleus; in the male pronucleus, the clusters orient themselves towards sperm centriole. The rays of the microtubule aster, which forms around the sperm centriole, reach the female pronucleus to direct the approach of the pronuclei to each other with the help of dynein. There is evidence that in a similar way in the contact of pronuclei, microtubules help pore complexes and chromatin to bound to dynein and to be directed to the centrosomes (Figure 2C,D). For human, bovine, and mouse zygotes, it has been found that during the polarization of chromatin clusters, NPCs are associated with chromosomal arms, but not with telomeres or centromeres [59,60].

### 3.6. Opening and Fusion of the Nuclear Envelopes of Pronuclei

After the meeting of the pronuclei, they are expected to unite, fusing their membranes, but this does not happen exactly like that. The parental genomes in pronuclei are separated by a total of four membranes, which makes the mechanism of unification very complex. However, in some animals such a union exists. In invertebrate models such as *Caenorhabditis elegans* [61], in the contact zone of the pronuclei in the nuclear envelopes, numerous extensive fenestrations appear. They allow the two genomes, performing semi-closed mitosis, to get closer to each other. Subsequently, the membranes of the pronuclei fuse through bonds that are similar to the three-way junctions in the formation of tubular endoplasmic reticulum. In some vertebrates, e.g., zebrafish (*Danio rerio*), there is also a fusion of the membranes of the pronuclei. It is carried out with the help of the transmembrane protein Brambleberry [62]. In some animals, during anaphase pronuclei are transformed into multiple micronuclei (karyomeres) containing separate chromatin clusters, that happens in sea urchin and fish zygotes, even in some mammals, such as rabbits. In these cases, Brambleberry is again responsible for the forming of karyomeres and their subsequent fusion into a common nucleus [63]. During human fertilization, female and male pronuclei juxtapose and break down synchronously. Before the breakdown the pronuclei stay juxtaposed for several hours. Finally, the nuclear envelopes interdigitate, disintegrate, and dissolve. Non-juxtaposition and asynchronous pronuclear breakdown leads to abnormal cleavage [64].

### 3.7. In the First Mitotic Spindle, the Maternal and Paternal Chromosomes Do Not Mix

**Figure 3:**
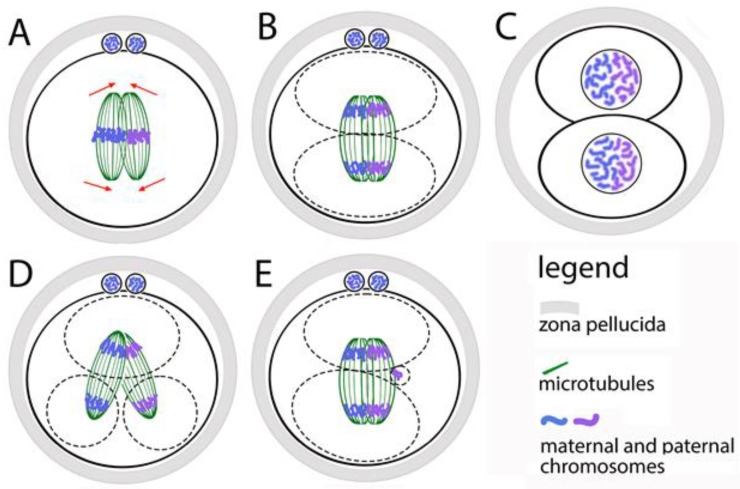
Formation of the first mitotic spindle of the zygote. (**A**) The maternal and paternal chromosomes are in separate spindles that must be aligned before anaphase occurs. (**B**) During the first division of the zygote, the maternal and paternal chromosomes do not mix. (**C**) In the two first blastomere nuclei, the maternal and paternal chromosomes stay in separate zones. (**D**) If the two spindles do not align, the zygote divides into more than two blastomeres with abnormal karyotype. (**E**) The anaphase lagging of single chromosomes leads to the appearance of additional micronuclei in the blastomeres.

After the first contact of the pronuclei and the formation of two centrosomes, the parental genomes are ready to participate in the first mitotic division of the zygote. In mouse zygotes, however, maternal and paternal chromosomes do not mix at this stage—instead of forming a common bipolar microtubular spindle, two bipolar spindles are formed in the zygote, each of which contains one parental chromosomal set [65] (Figure 3A). If we have to define at what stage of the cell cycle the two spindles are formed, the answer is early and middle prometaphase. The two spindles must stay parallel to each other and align their poles before the anaphase onset, but without mixing their chromosomes. Thus, after the division, in the nuclei of the first two blastomeres the maternal and paternal chromosomes occupy separate territories (Figure 3A–C). This was observed by microscopic analysis of zygotes and embryos, in which the centromeres of the maternal and paternal chromosomes are marked differently [66].

The functional independence of the two spindles is proven by the following facts: in prometaphase, when the chromosomes move to the equator (the process is denoted as chromosomal congression), the spindles are clearly separated; the congression of the two sets of chromosomes can be reached at different times, suggesting that they move from different systems of microtubules; the two chromosomal sets form two metaphase plates, which are located at an angle to each other and become parallel only at the final alignment of the spindles (Figure 3A,B). The maternal and paternal chromosomes are kept separate by two spindles only during the first mitosis. During the next divisions, they mix, because a single spindle is formed around the whole chromosomal set.

The described mechanism for assembling the first mitotic spindle leads to the risk of unsuccessful alignment of the two parental spindles before the first mitotic anaphase. This creates opportunities for abnormal karyokinesis, and therefore for errors in the karyotype of blastomeres (Figure 3D). Direct evidence of this is the fact that the increased distance between the two pronuclei (achieved by treatment with nocodazole), leads to a distance between the two spindles [66]. Most often, they fail to fully align their poles. This does not slow down anaphase but leads to the segregation of chromosomes from the two spindles in different direction. As a result, one or both blastomeres receive two nuclei. Zygotes that have successfully aligned the two spindles before anaphase divide normally into two mononucleated blastomeres.

The defects resulting from the failure to align the two spindles in mouse zygotes are very similar to defects described in human embryos obtained by in vitro fertilization. This suggests the idea that the described mechanism for assembling a double spindle is also valid for human zygotes, as well as for mammals in general. This hypothesis is also supported by the following facts: in clinics for assisted human reproduction, embryos with more than one nucleus in the blastomeres and with impaired chromosomal distribution are often observed [67]; in human zygotes, cases of separation of maternal and paternal chromosomes into separate groups have been described; in cattle cleaving embryos, blastomeres with only maternal, only paternal, as well as with mixed chromosomes have been seen [60].

### 3.8. Unusual Spatial Organization of Chromosomes in Gamete Nuclei and Preimplantation Embryos

The union of the two parental genomes after fertilization requires epigenetic changes. Several epigenomic studies have shown that chromatin changes in eggs, spermatozoa and early preimplantation embryos are not typical for any other stages—atypical histone modifications and unusual territorial organization of chromosomes have been observed. For example, in gamete chromosomes, topologically associated domains (TADs) are completely absent; they begin to form in the pronuclei of zygotes and gradually acquire their expected appearance as cell division progresses [65,68]. In an attempt to determine whether the parental features of chromosomal territories are inherited in the newly formed embryo and how they relate to allele-specific gene regulation, the interactions between all parental chromosomes (including the X chromosome) during the preimplantation development of a mouse were mapped [69]. The relationships between territorial chromosomal organization and allelic states and gene expression have been studied. It has been found that after fertilization, biased gene expression (only of maternal or paternal alleles) is observed in some chromosomal domains. In addition, some domains with active chromatin are observed in the embryonic but not in the parental genomes. For example, changes in the paternal X chromosome before and during inactivation of one of the X chromosomes in preimplantation female embryos have been traced [70]. It has been found that when certain genes are inactivated from the chromosomal territory, the corresponding territorially associated domains are removed.

### 3.9. Observations of Living Human Zygotes Confirm the Fact That the First Mitosis Is Unusually Long and Often Mistaken for Chromosomal Segregation

The movement and segregation of chromosomes during the first division of the zygote have been observed in living human zygotes by Currie and coauthors [71]. It has been found that the time from the destruction of the nuclear envelopes of the pronuclei to the arrangement of the chromosomes in a metaphase plate is two hours, and the time from the start of anaphase to the appearance of a dividing groove is about 45 min. These time periods are about 5 times longer than their corresponding ones in later embryonic cells and in ordinary somatic cells. In one-third of the living zygotes in this study, the following defects were observed: non-disjunction of sister chromatids; incorrect direction of chromosome movement; formation of two metaphase plates (which supports the hypothesis of a double spindle); anaphase lagging of chromosomes (in this case, delayed chromosomes are separated into micronuclei, the possible mechanism is shown at Figure 3E); formation of several chromosomal clusters at the poles of the spindle instead of one anaphase group, that form several nuclei instead of one (the possible mechanism is shown at Figure 3D).

### 3.10. Errors of the First Cytokinesis

It is expected that during the first division of the zygote, two identical blastomeres will be obtained. However, sometimes there is a division of three or more cells. In clinical practice, this is referred to as direct uneven cleavage or trichotomous cleavage (Figure 3D). This phenomenon was observed in more than 10% of the human IVF embryos [72]. The situation is similar in the second division, but in the third this is observed much less often—in about 1% of the embryos [73]. Trichotomous division leads to chaotic mosaicism in the blastomeres (presence of one or more trisomies, paternal and maternal monosomies and nullisomies, uniparental dysomies). Such chaotic mosaics are often observed in preimplantation genetic tests. Trichotomous cleavage is thought to be due to the formation of mitotic spindles with more than two poles in the blastomeres, which completely disturbs the formation of contractile rings of fibrillar actin during cytokinesis [74].

### 3.11. Principal Differences in the Occurrence of Chromosomal Abnormalities in Oocytes and Zygotes

The incidence of meiotic chromosomal errors in oocytes is unusually high, even in young women, and increases significantly with maternal age. Such errors lead to complete (uniform) aneuploidy in all cells of the resulting embryo. An incorrect distribution of chromosomes can occur both during meiosis I and during meiosis II. Uniform aneuploidy of early embryos is a leading cause of infertility, early miscarriages and birth defects.

In zygotes, a high level of errors in chromosomal distribution is also observed. It is significantly higher than the level of such errors in later mitotic divisions. Most often, irregular chromosomal segregation occurs at the first division of the zygote. Mitotic errors in early cleaving embryos lead to chromosomal mosaicism—aneuploidy in only some of the blastomeres, as well as various aneuploidy in the blastomeres of one embryo. No relationship has been observed between the mosaicism of early embryos and maternal age.

The table (Table 1) presented here summarizes how different cytoskeletal elements can be involved in abnormal chromosomal segregation in oocytes, zygotes, and blastomeres of early embryos.

Several research groups have shown that certain cytoskeletal stabilizers can improve outcomes in assisted reproductive technology, particularly by enhancing oocyte maturation. However, the effect on overall fertilization rates is complex and context-dependent. The appropriate type, concentration, and timing of stabilizer exposure are critical factors. Compounds like paclitaxel (Taxol) stabilize microtubules, helping to ensure the proper formation of the meiotic spindle [75]. Studies in mouse embryos suggest that treatment with low-dose paclitaxel can improve blastocyst development potential and decrease degeneration rates without affecting the aneuploidy rate [76]. Stabilizers can help protect the oocyte’s internal structure during the freezing and thawing process. The microfilament stabilizer cytochalasin B has been used during oocyte vitrification and warming to help the oocyte recover after thawing [77].

It should be borne in mind that factors other than those discussed here can also contribute to abnormal chromosomal segregation during meiosis, fertilization, and embryonic cell division. Errors in chromosomal distribution may be due to genetic and individual factors from both sperm and oocytes. Oocyte factors are maternal age, disturbances in chromosomal crossing over (insufficient number or poorly positioned crossovers), mitochondrial dysfunction, mutations in genes regulating oocyte-specific processes, environmental and lifestyle factors. Sperm factors that can disturb fertilization and embryonic cell division are sperm DNA fragmentation or other kinds of sperm DNA damage, aberrant chromatin packaging, centrosome abnormalities, lifestyle and environmental factors.

## 4. Conclusions

In conclusion, we can say that the unification of the maternal and paternal chromosomes as well as numerical chromosomal errors of mammalian oocytes and preimplantation embryos are largely due to the cytoskeletal problems:

Cytoskeletal defects that disturb oocyte meiosis (studied in rodent and primate oocytes [17,18,19,20,21,22,23,24,25,26,27,28,29,30,31,32,33,34,35,36,37,38,39,40,41,42,43,44,45]):

– Unsuccessful connection between cytoskeleton and nucleoskeleton by LINC;
– Unsuccessful arrangement of the small microtubular asters in a bipolar spindle;
– Unsuccessful movement of the spindle to the oocyte periphery;
– Unstable anchoring of the spindle to oolemma;
– Unsuccessful rotation of meiotic spindle for polar body extrusion;
– Inappropriate microtubular length;
– Unsuccessful attachment of kinetochores to microtubules;
– Deviations in meiotic spindle morphology.

Cytoskeletal defects that disturb pronuclear unification (studied in nematodes, rodent, cattle and human oocytes [11,46,47,48,49,50,51,52,53,54,55,56,57,58,59,60,61,62,63,64,66,67]):

– Nonfunctional male centriole, responsible for microtubule focusing;
– Unsuccessful forming of microtubule aster around the sperm centrosome;
– Unsuccessful capturing of female pronucleus by the sperm microtubule aster;
– Unsuccessful movement of the two pronuclei towards each other;
– Inappropriate contacts between centrosomes, dynein, microtubules, and nuclear pore complexes.

Cytoskeletal defects that disturb zygote mitosis (studied in rodent, cattle, and human [65,68,69,74,75,76,77]):

– Unsuccessful forming of bipolar mitotic spindle by alignment of the maternal and paternal parts;
– Non-synchronized congression of the two sets of chromosomes;
– Unsuccessful attachment of kinetochores to microtubules.

Future research on the contribution of cytoskeletal problems to chromosomal abnormalities in oocytes and early embryos can go in several directions. The latest advances in microscopic analyses of living single cells can reveal the ultrastructural dynamics of cytoskeleton and chromatin interactions in living eggs, zygotes, and embryos. Such studies will provide information on how chromatin-cytoskeletal interactions are related to oocyte aging. From future research, it is expected an answer to the question of whether indeed, as it seems, the human spindle in Meiosis I is inherently more unstable than in other species, and what is the reason for this. The data obtained will show which factors of in vitro cultivation can be manipulated to achieve higher success in assisted reproduction. Variations in the action of individual proteins in chromosome dynamics during meiosis, fertilization, and early embryogenesis would be a step toward studying the gene variants of these proteins. This would pave the way for the introduction of personalized medical approaches in patients with specific problems such as unexplained infertility, repeated miscarriages, and repeated unsuccessful IVF attempts.

## Figures and Tables

**Table 1:** Summarizes information about involvement of different cytoskeletal elements in abnormal chromosomal segregation in oocytes, zygotes, and blastomeres of early embryos.

Cytoskeletal Factor	Interacting Factor	Possible Mechanism to Contribute to Abnormal Chromosomal Segregation	Cell Type
Microtubules	LINC that connects nucleoskeleton and cytoskeleton	Incorrect synapse between homologous chromosomes	Oocytes
	Chromosomal arms	Incorrect attachment to chromosomes, resulting in misalignment or lag	Oocytes, zygotes, and blastomeres
	Kinetochores	Chromosomal misalignment or lag Disturbed control of the length of microtubules	Oocytes, zygotes, and blastomeres
	Kinesins and chromokinesins	Unsuccessful kinetochore-tubulin contacts Abnormal pole focusing Disorientation of spindle microtubules Unsuccessful congression of the chromosomes	Oocytes, zygotes, and blastomeres
	Sperm centriole	Unsuccessful movement of male and female pronuclei towards each other Unsuccessful arrangement of the first mitotic spindle	Zygotes
	Nuclear pores of the pronuclei	Abnormal contacts between microtubules and chromosomes Problematic movement of chromatin clusters towards the contact zone between the pronuclei	Zygotes
Nuclear intermediate filaments (lamins)	Microfilaments and chromatin	Disturbed contact between microtubules and chromosomes	Oocytes
Cytoplasmic intermediate filaments	Microfilaments and chromatin	Blocked opening of nuclear membranes Unsuccessful spindle arrangement	Oocytes
Microfilaments	Chromosomes	Unsuccessful spindle arrangement Blocked spindle movement to the oolemma	Oocytes
	Microtubules	Unsuccessful movement of the spindle to the oolemma Too large polar body	Oocytes
	Oolemma	Unsuccessful anchoring of the spindle to the oolemma Unsuccessful spindle rotation and polar body extrusion	Oocytes

## Data Availability

For the preparation of this review, no new data was created.

## Abbreviations

The following abbreviations are used in this manuscript:

ESHRE	European Society of Human Reproduction and Embryology
GV	Germinal Vesicle
IVF	In Vitro Fertilization
LINC	Linker of Nucleoskeleton and Cytoskeleton Complex
MPF	Maturation-promoting Factor
NEBD	Nuclear Envelope Breakdown
NPC	Nuclear Pore Complex
